# Correction to “The Big BIT maize experiment: A large multi‐location, multi‐year, multi‐tester, multi‐population predictive breeding validation study”

**DOI:** 10.1002/tpg2.70249

**Published:** 2026-05-14

Jines, M., Chandler, M., Podlich, D., Baumgarten, A., Wright, D., Jacobson, A., Zhao, H., Gogerty, J., Regennitter, M., Hung, H.‐Y., McDowell, R., Tang, T., Diepenbrock, C., Helmi, H., Messina, C., Ross, A., Henke, G., Spangler, L., Caldwell, M., … Totir, L. R. (2025). The Big BIT maize experiment: A large multi‐location, multi‐year, multi‐tester, multi‐population predictive breeding validation study. *The Plant Genome*, *18*, e70117. https://doi.org/10.1002/tpg2.70117

The Data Availability Statement has been updated. The previous statement read:

The data described and analyzed in this study were collected on maize plants that are part of Corteva's active multi‐billion‐dollar commercial maize breeding program. As such, the phenotypic and genomic data collected on these plants are confidential and protected as intellectual property or as trade secrets. As a result, the data described and analyzed in our study cannot be made publicly available but are stored in a secure Corteva database. Data can be made available on reasonable request by contacting the corresponding authors.

The last sentence of the above paragraph has been removed and replaced with new text:

The data described and analyzed in this study were collected on maize plants that are part of Corteva's active multi‐billion‐dollar commercial maize breeding program. As such, the phenotypic and genomic data collected on these plants are confidential and protected as intellectual property or as trade secrets. As a result, the data described and analyzed in our study cannot be made publicly available but are stored in a secure Corteva database. Further, the data were not made available during the peer‐review process due to their sensitive and proprietary nature. While the authors are working to obtain a formal mechanism by which the public may request the data, it is not available as of April 31, 2026.

